# Effect of different straw retention techniques on soil microbial community structure in wheat–maize rotation system

**DOI:** 10.3389/fmicb.2022.1069458

**Published:** 2023-01-19

**Authors:** Shulin Zhang, Meng Li, Xinyue Cui, Yuemin Pan

**Affiliations:** ^1^Department of Plant Pathology, College of Plant Protection, Anhui Agricultural University, Hefei, China; ^2^Anhui Province Key Laboratory of Crop Integrated Pest Management, Anhui Agricultural University, Hefei, China

**Keywords:** rotation system, soil microbial community, straw return, bacteria, fungi

## Abstract

Rotational straw return technique is considered an effective measure for improving soil quality and maintaining soil microorganisms. However, there are few reports on the influence of wheat–maize crop rotation and straw-returning tillage on crop soil microbial communities in China. This study aimed to investigate how wheat or maize straw-incorporation practices affect bacterial and fungal communities under wheat–maize rotational farming practices. To clarify the effects of straw incorporation on microbial composition, microbial communities from soils subjected to different treatments were identified using high-throughput sequencing. Our results showed that, before corn planting, wheat and maize straw returning reduced bacterial density and increased their diversity but had no effect on fungal diversity. However, before wheat planting, returning wheat and corn stalks to the field increased the diversity of soil bacteria and fungi, whereas returning corn stalks to the field reduced the diversity of fungi and other microorganisms. Straw return significantly increased the relative abundance of Ascomycota in the first season and decreased it in the second season; however, in the second season, wheat straw return increased the relative abundance of *Bradyrhizobium*, which can promote the soil microbial nitrogen cycle and provide nitrogen to the soil. Wheat and maize straw return increased the relative abundance of *Chaetomium*, whereas, individually, they decreased the relative abundance. In addition, we detected two fungal pathogens (*Fusarium* and *Trichoderma*) under the two planting patterns and found that the relative abundance of pathogenic Fusarium increased with wheat straw return (FW and SW). Trichoderma increased after treatment with maize straw return before wheat planting (S group). These results suggest that wheat straw return (FW and SW) and maize straw return might have a negative impact on the pathogenic risk. Therefore, further studies are needed to determine how to manage straw returns in agricultural production.

## Introduction

Soil microorganisms play an important role in the ecosystem and their diversity is a vital indicator of soil fertility and microecology (Habig et al., 2018). Soil microorganisms can interact with plants, especially through physically close areas such as roots and rhizosphere (Philippot et al., 2013). In addition, many soil microorganisms affect plant health. Bacteria and fungi are the predominant forms of ecologically significant microbial communities in soil, and previous studies showed that their presence has a significant impact on plant health or disease suppression (Bastida et al., 2008). Soil microbial community composition is affected by plant species, developmental stage, environmental factors, and management practices (Ma et al., 2016). Among other factors, management practices and farming methods are key to soil microbial communities (Zhang et al., 2019), and they may cause the re-emergence of soil-borne diseases (Nicola et al., 2017).

Anhui Province is one of the largest agricultural production regions in China, where winter wheat (*Triticum aestivum* L.) and summer maize (*Zea mays* L.) are major crops (Lu et al., 2022). In this region, winter wheat and summer maize rotation is the common cropping pattern (Su et al., 2020). Wheat and maize straw is burned immediately after harvest, which not only wastes resources but also causes serious environmental pollution. Straw return is widely applied in rotation systems to prevent the impact of straw burning on the environment. The incorporation of straw into soil has become a key measure for improving soil quality (Yu et al., 2020). Therefore, returning straw to the field was often an effective measure for protecting the environment and maintaining the soil microbial community characteristics (Marschner et al., 2011; Yang et al., 2019). However, straw return is usually deemed as the main causal agent of soil-borne diseases, such as wheat scab, wheat crown rot, corn northern leaf blight, southern leaf blight, and maize ear rot, as it provides good circumstances for pathogen growth, propagation, and accumulation, which then results in disease epidemics (Zhen et al., 2009; Li et al., 2012; Qiao et al., 2013). At present, there have been many reports on the impact of different straw-returning methods on the soil microbial community structure. Tang et al. (2020) found that wheat straw retention increased the fungal community diversity. Compared to the control group, the relative abundance of some bacteria, including Actinobacteria, Chloroflexi, and Saccharibacteria, increased with wheat straw addition. Regarding fungi, the relative abundance of *Fusarium* decreases with the addition of wheat straw (Tang et al., 2020). However, Yang et al. (2019) found that wheat straw return significantly affected the α-diversity of the soil bacterial community but not the fungal community. It enhanced the relative abundance of Proteobacteria and Zygomycota but reduced that of Acidobacteria and Ascomycota (Yang et al., 2019).

Although a number of studies showed that crop rotation systems have positive effects on soil microbial communities, there are few reports on the influence of wheat–maize crop rotation and straw-returning tillage on the crop rhizosphere soil microbial community in Anhui Province, China. Therefore, understanding the soil microbial community under these agricultural practices would have a positive effect on agricultural development. In this study, we collected 24 soil samples from eight types of straw-returning tillage under a wheat–maize crop rotation system, including both wheat and maize straw return, wheat straw return, maize straw return, and no-straw return before maize (First season, F) or wheat (Second season, S) planting. To understand the soil microbial community of the various straw-returning tillage systems, we performed bacterial 16S rRNA and fungal ITS amplicon sequencing of these samples and analyzed the sequencing data. Our study aimed to analyze the soil microbial communities after different treatments, which could provide a scientific basis for the role of straw return in agricultural practices.

## Materials and methods

### Description of the study area and materials

The experimental field was located in Mengcheng, Anhui Province (32°55′-33°29′N, 116°15′-116°49′E). This location has a warm temperate and sub-humid continental monsoon climate. The region has an annual average temperature of 14.8°C and a mean annual precipitation of 732.63 mm, mainly in June, July, and August. Moreover, this region has an annual frost-free period of 214 days, with an average sunshine duration of 2,410 h, a relative humidity of 72%, and an optimum temperature period from March to June and from September to November. The experiment was conducted for 2 years and was divided into two seasons in 2021 based on the wheat–maize rotation system. There were eight treatments with three replicates before maize or wheat planting. FB, FCK, FM, and FW represent wheat and maize straw, no straw, maize straw, and wheat straw retention in the field before maize planting, respectively. SB, SCK, SM, and SW represent wheat and maize straw, no straw, maize straw, and wheat straw retention in the field before wheat planting, respectively. F represents the season of wheat planting before corn planting and S represents the season of corn planting before wheat planting.

### Soil sampling and analysis

Soil samples were collected from fields with wheat and maize straw return (FB and SB), maize straw return (FM and SM), wheat straw return (FW and SW), and no straw return (FCK and SCK) under a long-time wheat–maize rotation system. A total of 8 treatment plots and 24 soil samples were taken. Before sampling, sand and gravel were removed from the soil samples. The sampling point (the distance between each sampling point was <100 m) was a square with a side length of 20 cm. Soil from the marked locations was excavated with a soil shovel (using the five-point sampling method); 0–20 cm (~30 g) of shallow soil was collected from each sampling point and fully mixed. Then, the soil samples were sieved using a 2-mm pore-size filter (Sun et al., 2020). Each collected soil sample was placed in a 50-ml sterile centrifuge tube, marked, and stored in an ice box. After air drying, the collected soil samples were stored at −80 °C. All soil samples were air-dried and fully mixed after removing the pebbles and plant residues.

### DNA extraction and PCR amplification

Microbial DNA was extracted from each of the 24 samples using a FastDNA Spin Kit for soil (MP Biomedicals, Santa Ana, CA, United States), according to the manufacturer's protocols (Sun et al., 2022). The DNA quality and quantity were determined using a NanoDrop 2000 spectrophotometer (Bio-Rad Laboratories Inc., USA.). The V4–V5 region of the bacteria 16S ribosomal RNA gene and the ITS gene was amplified by PCR (95°C for 5 min, followed by 25 cycles at 95°C for 30 s, 30 s at ${\text{T}}_{\text{m}}^{{}^{\circ}}$C, and 72°C for 45 s, and a final extension at 72°C for 10 min and 10°C until halted by user) using the primers 515F 5′-GTGCCAGCMGCCGCGG-3′, 907R 5′-CCGTCAATTCMTTTRAGTTT-3′, ITS1F 5′-CTTGGTCATTTAGAGGAAGTAA-3′, and ITS2R 5′-GCTGCGTTCTTCATCGATGC-3′, where the barcode is an eight-base sequence unique to each sample. The PCR reactions were performed in triplicate in a 20 μl of a mixture containing 4 μl of 5× FastPfu Buffer, 2 μl of 2.5 mM dNTPs, 0.8 μl of each primer (5 μM), 0.4 μl of FastPfu Polymerase, and 10 ng of template DNA. The amplicons were extracted from 2% agarose gels, purified using an AxyPrep DNA Gel Extraction Kit (Axygen Biosciences, AP-GX-4) according to the manufacturer's instructions, and quantified using QuantiFluor™-ST (Promega, US).

### Library construction and sequencing

The purified PCR products were quantified by Qubit^®^3.0 (Life Invitrogen) and every 24 amplicons with different barcodes were mixed equally. The pooled DNA product was used to construct an Illumina pair-end library following the Illumina genomic DNA library preparation procedure. The amplicon library was pair-end sequenced (2×250) using an Illumina MiSeq platform (Shanghai BIOZERON Co., Ltd.), according to standard protocols. The raw reads were deposited onto the NCBI Sequence Read Archive (SRA) database with an accession number PRJNA898898.

### Bioinformatic analysis

Raw fastq files were first demultiplexed using in-house Perl scripts according to the barcode sequence information for each sample with the following criteria: (i) the 250 bp reads were truncated at any site receiving an average quality score of <20 over a 10 bp sliding window, discarding the truncated reads that were shorter than 50 bp. (ii) exact barcode matching, two nucleotide mismatches in primer matching, and reads containing ambiguous characters were removed; and (iii) only sequences that overlapped by more than 10 bp were assembled according to their overlap sequence. The reads that could not be assembled were discarded.

Operational taxonomic units (OTUs) were clustered with a 97% similarity cutoff using UPARSE version 7.1 (http://drive5.com/uparse/), and chimeric sequences were identified and removed using UCHIME. The phylogenetic affiliation of each 16S rRNA gene sequence was analyzed by the UCLUST algorithm (http://www.drive5.com/usearch/manual/uclust_algo.html) against the SILVA (SSU138.1) 16S rRNA database using a confidence threshold of 80% (Amato et al., 2013). The phylogenetic affiliation of the ITS gene sequence was analyzed by the UCLUST algorithm (http://www.drive5.com/usearch/manual/uclust_algo.html) against Unite (Release 7.1) using a confidence threshold of 80% (Koljalg et al., 2013).

### Statistical analysis

The rarefaction analysis based on Mothur v.1.21.1 (Schloss et al., 2009) was conducted to reveal the diversity indices, including the Chao, ACE, and Shannon diversity indices. The beta diversity analysis was performed using UniFrac (Lozupone et al., 2011) to compare the results of the principal coordinates analysis (PCoA) using the community ecology package (Vegan 2.0). A one-way analysis of variance (ANOVA) was performed to assess the statistically significant differences among diversity indices between samples. The differences were considered significant at a *P* < 0.05. The Venn diagrams were drawn using the online tool “Draw Venn Diagram” (http://bioinformatics.psb.ugent.be/webtools/Venn) to analyze the overlapped and unique OTUs during the treatment processes. A one-way permutational analysis of variance (PERMANOVA) was performed using the R vegan package to assess the statistically significant effects of the treatment processes on bacterial communities.

To identify biomarkers for high-dimensional colonic bacteria, the linear discriminant analysis effect size (LEfSe) was used (Segata et al., 2011). The Kruskal–Wallis sum-rank test was performed to examine the changes and dissimilarities among classes, followed by LDA analysis to determine the size effect of each distinctively abundant taxa (Ijaz et al., 2018). The biomarkers with significant differences were found by a random forest analysis using the “randomForest” package in R. The “igraph” package was used to analyze the cooccurrence network of the bacterial community structure, and Gephi 0.9.2 software was used to draw the network map.

## Results

### Effects of crop straw return on the α-diversity of soil bacterial and fungal communities

**Figure 1:**
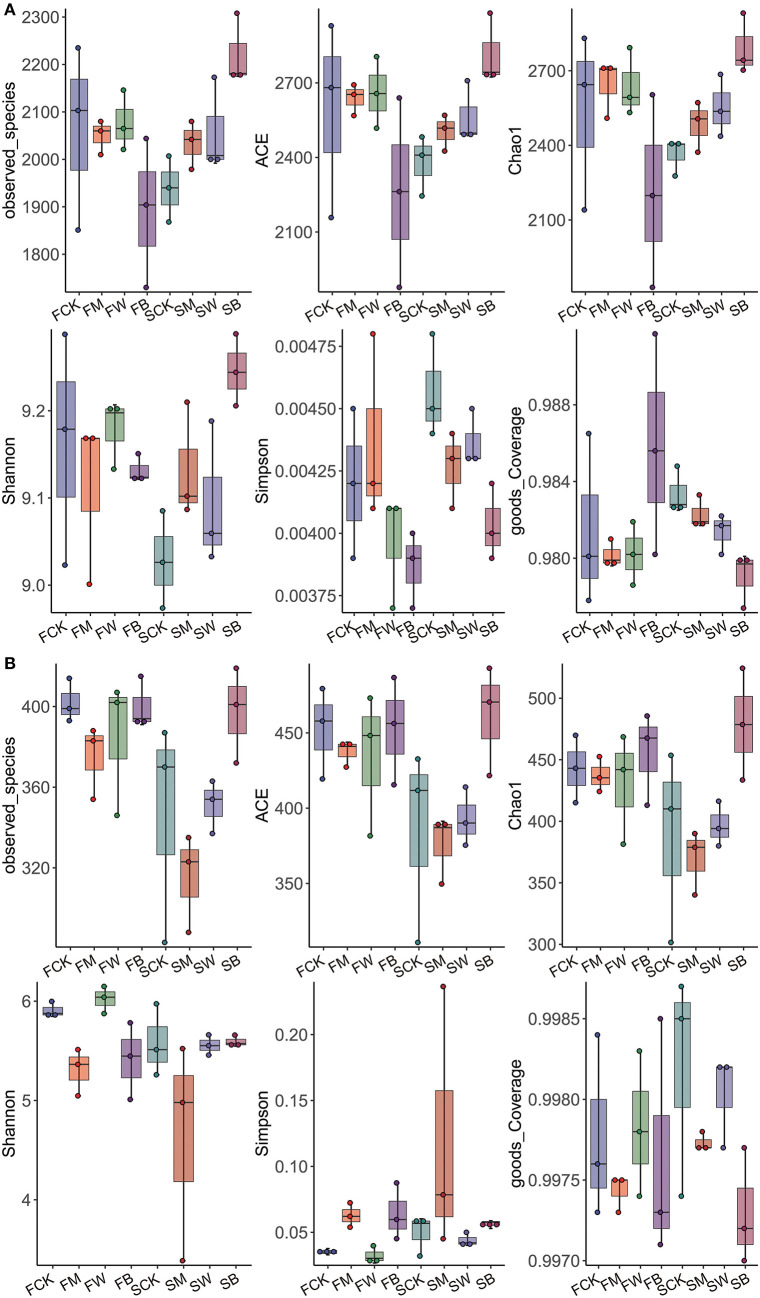
α-diversity of bacteria and fungi among the eight treatments during the two seasons. α-diversity of bacterial microorganisms in the soil **(A)**. α-diversity of fungal microorganisms in the soil **(B)**.

According to the bacterial microbial soil diversity analysis results (Figure 1A), there were no significant differences in the Chao, ACE, and Shannon indices between the FM and FW groups compared with the FCK group, whereas the FB group was significantly lower than the control group, indicating that, before corn planting, the wheat and maize straw return mode significantly changed bacterial diversity in soil and showed a downward trend. Compared with the SCK group, the Chao, ACE, and Shannon indices of the SM, SW, and SB groups were significantly higher than those of the control group, and the SB group reached the highest value, indicating that, before wheat planting, both corn return and wheat return significantly increased the diversity of bacteria and microorganisms in the soil, particularly when wheat and corn were returned together with straw. We also analyzed the diversity of fungal microorganisms in the soil (Figure 1B). The results showed that, compared with the FCK group, the Chao, ACE, and Shannon indices of the FM, FW, and FB groups were not significantly different. The Chao and ACE indices of the SB group were significantly higher than those of the SCK group, indicating that the straw return modes before corn planting did not significantly affect the diversity of soil fungal microorganisms, whereas the wheat and maize straw return mode before wheat planting increased the diversity. The Chao and Shannon indices of SM were lower than those of SCK, indicating that maize straw return reduced the soil fungal microbial diversity.

In summary, we found that, before corn planting, wheat and corn straw return increased bacterial diversity and had no effect on fungal diversity. However, before wheat planting, the straw-returning mode increased the diversity of bacteria in the soil and did not affect the diversity of fungi. Straw return before different crops has different effects on soil microorganisms. This conclusion provides a scientific reference for the rational use of straw returns.

### Venn analysis of the soil microbial community upon crop straw return

**Figure 2:**
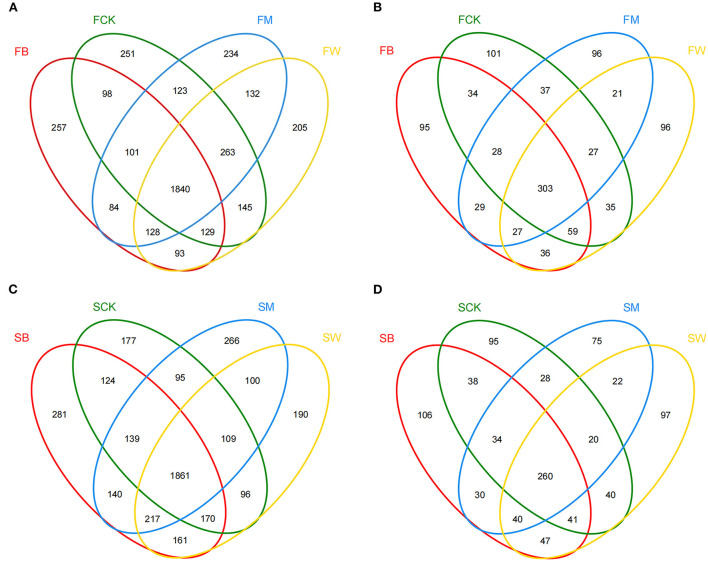
Venn diagrams of OTU distribution of the 16S rRNA gene **(A, C)** and the ITS gene **(B, D)** among the eight treatments in two seasons.

A Venn diagram was used to count the number of OTUs shared and unique among multiple samples. Before maize planting, the number of bacterial OTUs shared between different crop straw return treatments was 1,840 (45.1%), which was higher than the number of shared fungal OTUs, which was 303 (29.6%) (Figures 2A, B). The number of unique bacterial OTUs in the FB, FCK, FM, and FW groups was 257, 251, 234, and 205, respectively. In addition, the number of unique fungal OTUs of the FB, FCK, FM, and FW groups was 95, 101, 96, and 96, respectively (Figures 2A, B). Before wheat planting, the number of OTUs shared between different straw return techniques was 1,861 (45.1%) for bacteria and 260 (26.7%) for fungi (Figures 2C, D). The number of unique bacterial OTUs of the SB, SCK, SM, and SW groups was 281, 177, 266, and 190, respectively. However, the number of unique fungal OTUs of the SB, SCK, SM, and SW groups was 106, 95, 75, and 97, respectively (Figures 2C, D). These results showed that the bacterial OTU composition was higher than the fungal OTU composition in all samples.

### Effect of crop straw return on soil microbial community β-diversity

**Figure 3:**
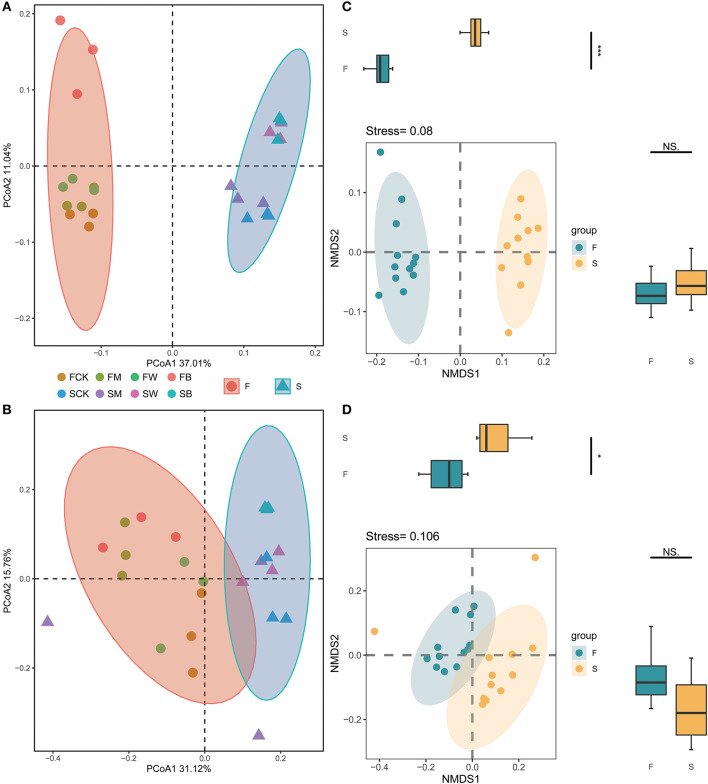
Effect of crop straw return on soil microbial community β-diversity. Principal coordinate analysis (PCoA) of the bacterial 16S rRNA gene **(A)** and fungal ITS gene **(B)** communities subjected to different treatments and nonmetric multidimensional scaling (NMDS) of the bacterial 16S rRNA gene **(C)** and the fungal ITS gene **(D)** communities subjected to different treatments. Each colored and shaped dot represents the sample.

The differences in the compositions of the microbial communities among the samples and between the groups of samples (β-diversity) were analyzed by using principal coordinate analysis (PCoA) or non-metric multidimensional scaling (NMDS). In the entire dataset, 534,561 bacterial and 509,081 fungal sequences were obtained from 24 soil samples, which were divided into 8,475 and 2,044 OTUs, respectively. The PCoA analysis showed that, for bacterial and fungal communities, there were significant differences in the overall composition of soil microorganisms between Groups F and S, in which PC1 explained 37.01% and 31.12% of the variations, respectively, and PC2 explained 11.04% and 15.76% of the variations, respectively (Figures 3A, B). These results also showed that, compared with the control group, the soil microbial compositions of the different treatment groups, such as FB, FW, SB, and SW, showed significant differences, indicating that different straw returning would lead to significant separation of bacterial and fungal microbial compositions in the soil, resulting in significant differences in the structure of the bacterial and fungal communities in the soil. We also analyzed the community compositions of the F and S groups based on NMDS (Figures 3C, D). The statistical results showed that the overall microbial compositions of groups F and S were significantly different.

### Microbial community structure and composition

**Figure 4:**
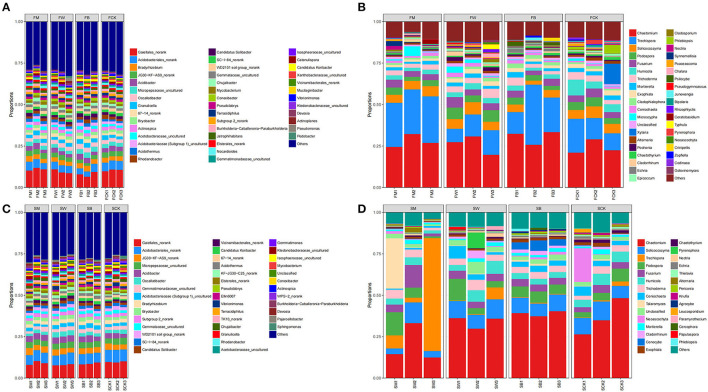
Microbial composition at the genus level. The 16S rRNA gene-based bacterial **(A)** and the ITS gene-based fungal **(B)** community compositions at the genus level subjected to four different treatments in the F group (to achieve the best view effect, the parts with abundance <1% can be merged with others in the diagram). F represents the season before the maize planting. The 16S rRNA gene-based bacterial **(C)** and the ITS gene-based fungal **(D)** community compositions at the genus level after the four treatments in the S group (to achieve the best view effect, the parts with abundance <1% can be merged in the diagram). S represents the season before wheat planting.

The analysis of the microbial community structure showed that, before maize planting (the F group), proteobacteria was the most dominant bacterial phylum in all samples, comprising 29.32% of the total sequences on average. The other dominant phyla were Actinobacteriota (27.23%), Acidobacteriota (19.85%), Chloroflexi (7.07%), and Myxococcota (1.20%). Regarding fungal sequences, Ascomycota was the dominant phylum level in all samples, comprising 68.74% of the total sequences on average. Other dominant phyla included Basidiomycota (27.27%), Mucoromycota (2.30%), and Chytridiomycota (1.25%) (Supplementary Tables S1, S2). In addition, at the phylum level, FW and FM significantly increased the relative abundance of Actinobacteriota (*P* < 0.05). FB significantly decreased the relative abundance of Actinobacteriota. In contrast, FM and FW significantly decreased the relative abundance of Acidobacteriota, whereas FB significantly increased the relative abundance of Acidobacteriota (*P* < 0.01). FM increased the relative abundance of Chloroflexi, whereas FW and FB decreased the relative abundance. Both FW and FB significantly increased the relative abundance of Myxococcota, whereas FM significantly decreased the relative abundance (*P* < 0.05). Regarding fungal communities, only FW significantly increased the relative abundance of Ascomycota, whereas FM and FB decreased it (*P* < 0.05). Both FM and FB significantly increased the relative abundance of Basidiomycota, whereas FW decreased it (*P* < 0.05) (Figure 4A; Supplementary Figure S1). At the genus level, the relative abundance of Gaiellales_norank (*P* < 0.05) and JG30-KF-AS9Norank (*P* < 0.01) were significantly increased in FM, whereas they were significantly decreased in FW and FB. The relative abundance of *Bradyrhizobium* increased both in FM and FB but decreased in FW. The relative abundance of *Occallatibacter* significantly decreased in FM, whereas FW and FB showed increased abundance. Regarding fungal communities, the relative abundances of *Chaetomium* and *Cladophialophora* were increased in the FM, FW, and FB groups (*P* < 0.05). The relative abundances of *Podospora, Fusarium, Trichoderma*, and *Exophiala* increased in FW but decreased in FM and FB. In addition, the relative abundance of *Humicola* and *Mortierella* decreased in FM, FW, and FB (Figure 4B; Supplementary Figure S1).

Before wheat planting (S group), Proteobacteria was the most dominant bacterial phylum in all samples, comprising 29.50% of the total sequence on average. Other dominant phyla were Acidobacteriota (22.50%), Actinobacteriota (18.31%), Chloroflexi (8.46%), and Planctomycetota (6.92%). At the same time, Ascomycota was the most dominant phylum, comprising 75.65% of the total sequences on average. Basidiomycota (19.61%) and Mucoromycota (1.75%) were the other dominant phyla (Supplementary Tables S3, S4). The above results showed that there were differences in the bacterial and fungal community structures among the four treatments.

We found that the relative abundance of Proteobacteria increased in SW, SM, and SB at the phylum level, and the relative abundance of Acidobacteriota and Myxococcota also increased in SW and SB (*P* < 0.05). The relative abundance of Actinobacteriota was increased in SM, whereas it decreased in SW and SB. As with maize straw returning, the relative abundance of Chloroflexi decreased in SW and SB, whereas the relative abundance of Myxococcota significantly increased. Regarding the fungal community, the relative abundance of Ascomycota significantly decreased in both SM and SB (Figure 4C; Supplementary Figure S2). At the bacterial community genus level, the relative abundance of Gaiellales_norank and Acidobacteriales_norank significantly decreased, whereas the relative abundances of JG30-KF-AS9 norank, *Acidibacter, Bradyrhizobium*, and *Bryobacter* increased but not significantly in SM, SW, and SB. In the fungal community, the relative abundance of *Chaetomium* increased in SB but decreased in SM and SW. In addition, the relative abundances of *Solicoccozyma, Humicola*, and *Coniochaeta* significantly increased in SW, whereas they decreased in SM and SB. The relative abundances of *Podospora, Fusarium*, and *Trichoderma* increased in SM, SW, and SB. The relative abundance of *Talaromyces* increased in SM and SB but decreased in SW (Figure 4D; Supplementary Figure S2). The above results indicate that different straw-returning methods specifically promote the proliferation of bacteria or fungal microorganisms in certain soils, thereby changing their relative abundance.

**Figure 5:**
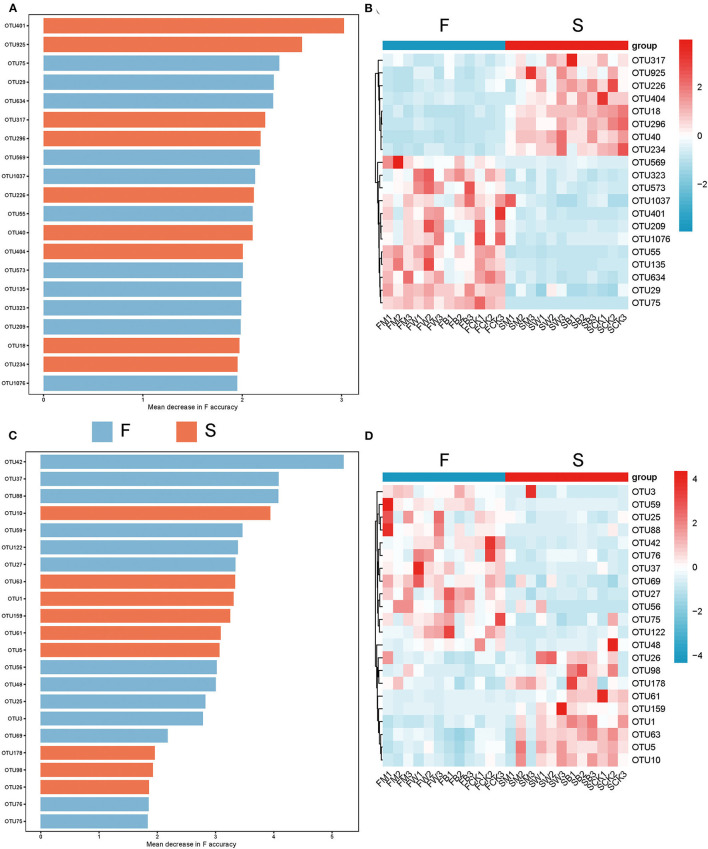
Microbes with the best identification ability screened from random forests under different straw-returning modes. **(A, B)** The 20 most discriminant genera in the models classifying the F and S group bacterial microorganisms at the genus level. The bar lengths indicate the importance of the variable, and the colors represent enrichment in F (green shades) or S (red shades). **(C, D)** The 22 most discriminant genera in the models classifying the F and S group fungal microorganisms at the genus level. The bar lengths indicate the importance of the variable and the colors represent enrichment in F (green shade) or S (red shade).

To explore the differences in the biomarkers between these groups, we developed a random forest model based on differential OTU with a relative abundance of >0 in at least 95% of samples from the F and S groups. The optimal model utilized 20 genera that provided the best discriminatory power (Figure 5A). These genera in the optimal model were primarily members of OTU 401 (corresponding to Intrasporangiaceae), OTU 925 (corresponding to Myxococcaceae), etc. At the same time, we found from the corresponding heatmap that the relative abundance of a large number of microorganisms at the genus level in the F group showed a significant downward trend (Figure 5B), such as OTU 317 (corresponding to Acetobacteraceae). The above results showed that straw returning before planting different crops will cause a sharp decline in a large number of specific bacteria, inhibit the proliferation of some bacterial microorganisms, and significantly change the dominant flora, which is also consistent with the results described above.

We also used the random forest model to explore the biomarkers of the fungal microorganisms in the F and S groups at the genus level. The optimal model utilized 22 genera that provided the best discriminatory power (Figure 5C). These genera were primarily the members of OTU 42 (corresponding to *Alternaria*), OTU 37 (corresponding to Pochonia), etc. At the same time, we found that the F group also significantly reduced the relative abundance of a large number of fungal flora (Figure 5D), such as OTU 26 (corresponding to Chaetomium) and OTU 98 (corresponding to Otidea), and straw returning before planting different crops also significantly inhibited the proliferation of a large number of fungal microorganisms, resulting in a significant decline in their abundance and selectively promoting the relative abundance of dominant fungi, which promoted the transformation of dominant fungi.

### Effect of crop straw return on bacterial and fungal biomarkers

**Figure 6:**
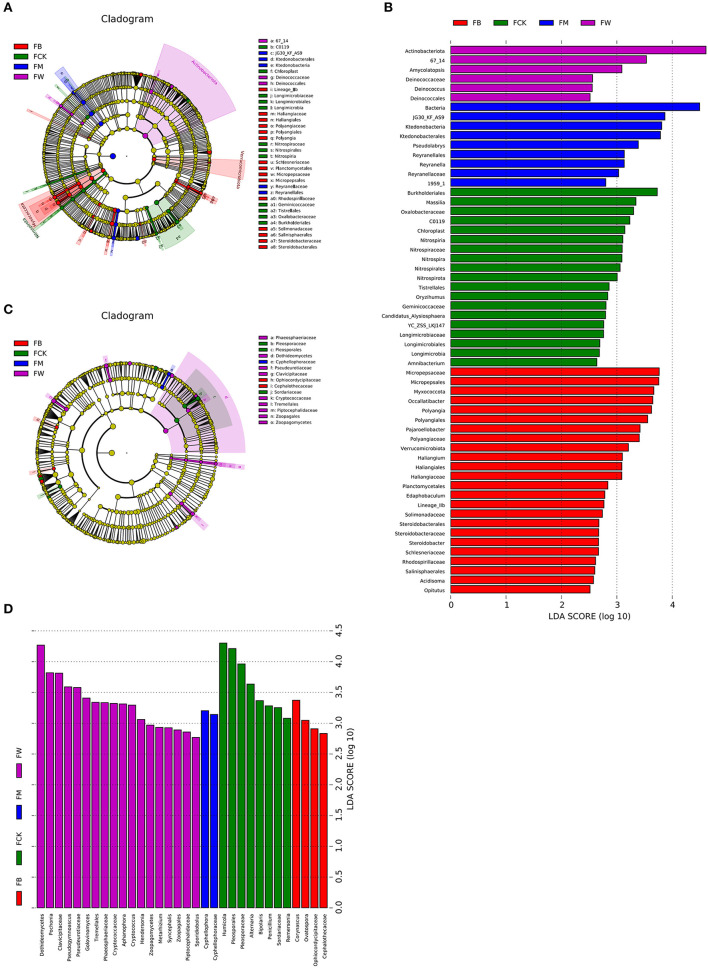
Cladograms plotted from the LEfSe analysis show significant differences (*P* < 0.05) in the relative abundance of 16S rRNA gene-based bacterial taxa among the four treatments before maize planting **(A)**. The results of LEfSe analysis show taxa that differed significantly among the four treatments before maize planting **(B)**. The cladogram plotted from LEfSe analysis shows significant differences (*P* < 0.05) in the relative abundance of the ITS gene-based fungal taxa among the four treatments **(C)**. The results of LEfSe analysis show taxa that differed significantly among the four treatments **(D)**.

Linear discriminant analysis effect size (LEfSe, Wilcoxon, *P* < 0.05, LDA score >2) was also used to identify the bacterial and fungal community biomarkers under different treatments. Apart from the difference at the phylum and genus levels, there were also other groups of microorganisms (bacteria and fungi) that were enriched in the four treatments and identified at the class, order, family, and genus levels. Two groups of bacteria, 67_14 and Deinococcaceae (from order to family), were significantly enriched in FW. A total of 10 groups of bacteria, including Micropepsaceae, Polyangiales, Lineage Iia, Haliangiaceae, Schlesneriaceae, Planctomycetales, Rhodospirillaceae, Solimonadaceae, Salinisphaerales, and Steroidobacteraceae, were significantly enriched in FB. Three groups of bacteria, JG30_KF_AS9, Ktedonobacteria, and Reyranellaceae, were significantly enriched in FM. Eight groups of bacteria, Burkholderiales, Oxalobacteraceae, Nitrospiraceae, Longimicrobiaceae, c0119, chloroplast, Geminicoccaceae, and Tistrellales, were significantly enriched in FCK (Figures 6A, B). Eight groups of fungi, phaeosphaeriaceae, Dothideomycetes, Pseudeurotiaceae, Cryptococcaceae, Tremellales, Piptocephalidaceae, and zoopagales, were significantly enriched in FW. Two groups of fungi were significantly enriched in FB, namely Ophiocordycipitaceae and Cephalothecaceae. Only one group of fungi, Cyphellophoraceae, was significantly enriched in FM. Pleosporaceae (from order to family) and Sordariaceae were the two groups of fungi that were significantly enriched in FCK (Figures 6C, D). A total of 10 groups of bacteria were significantly enriched in SW, namely Acidobacteriaceae_Subgroup_1_, SBR1031, Lineage_lla, mle1_27, Cpla_3_termite_group, Phycisphaerae, Schlesneriaceae, Comamonadaceae, JG36_TzT_191, and KF_JG30_C25. Seven groups of bacteria were significantly enriched in SM, namely Actinospicaceae, Blastocatellaceae (from class to family), Nocardioidaceae, Propionibacteriales, S085, Dehalococcoidia, and OLB14. A total of 11 groups of bacteria were significantly enriched in SB, namely Koribacteraceae, Frankiales, env_OPS_17, 0319-6G20, Oligoflexia, Candidatus_Azambacteria, Saccharimonadales (from class to order), Elsteraceae, TAR3_20, R7C24, and eub62A3. Thermoleophilia and Cyanobacteriales were the two groups of bacteria that were significantly enriched in SCK, namely (Supplementary Figures S3A, B).

Five groups of fungi were significantly enriched in SW, namely, Phaeosphaeriaceae, Hoehnelomycetaceae, Atractiellales (from class to order), Cryptococcaceae, and Tremellales. No fungi were significantly enriched in SM. Six groups of fungi were significantly enriched in SB, namely, Myxotrichaceae, Orbiliales (from class to order), Bionectriaceae, Lachnocladiaceae, Russulales, and Glomeromycetes. Seven groups of fungi were significantly enriched in SCK, namely, Sympoventuriaceae, Venturiales, Aspergillaceae, Pseudeurotiaceae, Cephalothecaceae, Rhizopodaceae, Mucorales, and Mucoromycetes (Supplementary Figures S3C, D). These results indicated that different straw returns have different bacterial and fungal biomarkers. Understanding the compositions of bacteria and fungi that are specifically enriched by different straw returns is an important reference for comprehensively understanding the promotion and harmful effects of soil.

### Effect of crop straw return on co-occurrence network relationship

**Figure 7:**
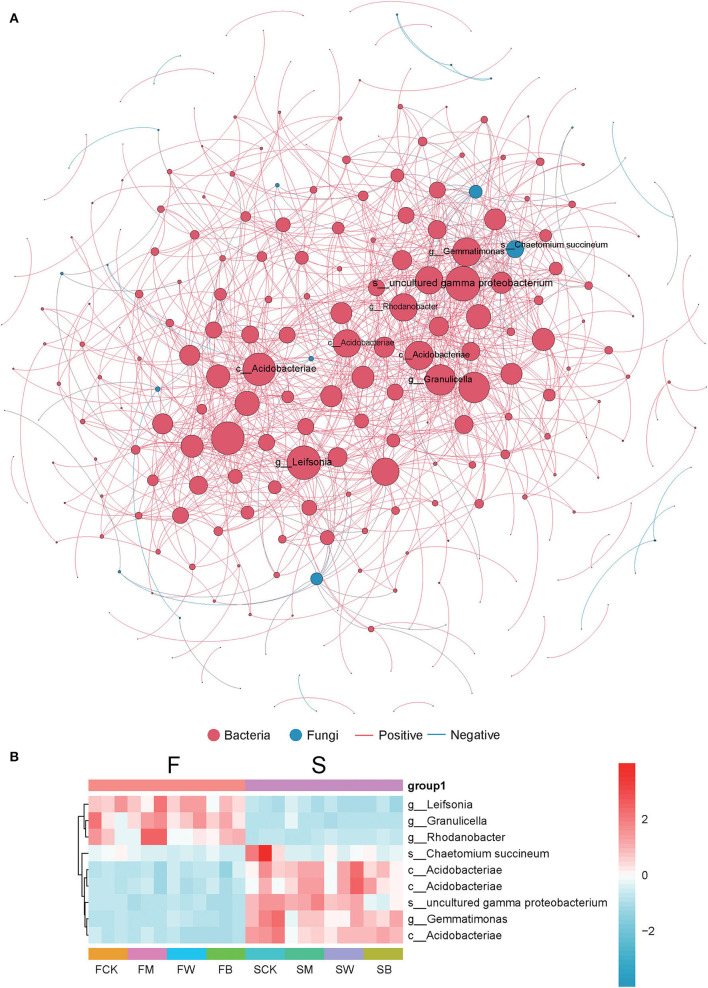
Co-occurrence network analysis of soil microorganism communities. The size of the node is proportional to the relative abundance of the genus. Each connection shown has a Spearman's coefficient of >0.6 and a *P* < 0.05. The red color indicates bacteria, the green color indicates fungi, the red line shows a positive correlation, and the blue line shows a negative correlation **(A)**. The heat map of several abundant dominant bacteria in a collinear network **(B)**.

To further explore the changes in soil colonies, we analyzed the symbiotic network relationship between the F and S groups based on Spearman's correlation coefficient. In Figure 7A, we found that bacteria dominate the symbiotic relationship among soil microorganisms under different straw-returning models and are the main components of the symbiotic systems. The dominant bacteria included Gemmatimonas, gamma proteobacterium, Rhodanobacter, and Leifsonia. *Leifsonia, Granulicella*, and *Rhodanobacter* are highly abundant in the F group than in the S group, while *uncultured gamma proteobacterium, Gemmatimonas*, and Acidobacteriae were abundant in the S group (Figure 7B).

## Discussion

In this study, high-throughput sequencing technology was used to study soil microbial communities subjected to different straw return patterns in Mengcheng County, Anhui Province. We mainly focused on the bacterial and fungal communities and found that straw return had an effect on soil microbial community diversity compared with no return (FCK and SCK). Before corn planting, wheat and corn straw returning reduced the abundance of bacterial microorganisms and increased their diversity. However, before wheat planting, wheat and maize straw return increased the abundance and diversity of bacterial microorganisms in soil and increased the abundance of fungal microorganisms; however, maize straw return reduced soil fungal microbial diversity. The results of Su et al. (2020) were consistent with those of our study. They found that, compared with the wheat straw return, the maize straw return showed a lower diversity of fungal communities and abundant fungal pathogens (Leptophaeria). Therefore, they suggested that maize straw return negatively impacts the diversity of soil fungal communities and the risk of disease, which may be related to changes in soil electrical conductivity. However, Guo et al. (2022) studied the impact of wheat straw return on improving soil microorganisms, quality, and crop yield and found that it improved soil microbial activity and bacterial species richness and diversity in deep soil layers.

Furthermore, PCA and NMDS analysis showed that the soil microbial community structures of wheat and maize were affected by straw returning. Therefore, we further analyzed the specific compositions of soil bacterial and fungal microorganisms subjected to different straw-returning modes to explore their impact on soil microorganisms. An analysis of the composition of soil microbial communities showed that straw returning had a significant effect on the composition of the soil microbial community. The dominant bacterial phyla detected in wheat–maize soil were Proteobacteria, Actinobacteriota, Acidobacteriota, and Chloroflexi in both seasons. We also found beneficial genera in the bacterial community such as *Bradyrhizobium*, a nitrogen-fixing rhizobacterium that provides nitrogen to the soil and thus promotes crop growth (Gan et al., 2015; Liu et al., 2020).

The diversity of the bacterial and fungal communities changes in response to the tillage systems and management measures (Li et al., 2012; Samaddar et al., 2019). In this study, first- and second-season straw-returning methods had different effects on the soil microbial community. In the first season, FW and FB significantly increased the relative abundance of Myxococcota, whereas FM significantly decreased it. FW significantly increased the relative abundance of Ascomycota. The relative abundance of *Bradyrhizobium* increased in the order FB > FM > FW > FCK. In the second season, the order of the relative abundance of *Bradyrhizobium* was SW > SCK > SM > SB. In other words, compared with no straw return (SCK), only wheat straw return increased the relative abundance of bacterial genera in the soil, whereas maize straw return and both wheat straw and maize straw return decreased the relative abundance of bacterial genera in the soil. Yang et al. (2019) studied the effect of wheat straw return on soil bacterial and fungal communities using a wheat soybean rotation system in a 2-year field study. The results showed that wheat straw return significantly affected α-diversity, enriching the relative abundance of nitrogen-cycling bacteria, such as *Bradyrhizobium* and *Rhizobium*. This conclusion is consistent with our results. At the same time, their preliminary analyses of soil chemical properties showed that the total nitrogen (TN) content of straw-returning soil was significantly higher than that of non-straw-returning soil. These results indicate that wheat straw return may promote the nitrogen cycle of soil microorganisms and play an important role in their physical and chemical properties.

In fungal communities, Ascomycota, Basidiomycota, and Mucoromycota were the dominant fungi in wheat and maize soils, which is consistent with the results of previous studies (Bai et al., 2015). The increasing relative abundance of Ascomycota may promote the degradation of organic matter (Schoch et al., 2009; Zhao et al., 2021). Guo et al. (2022) studied the impact of straw on the physical and chemical properties of soil and the diversity of the fungal community structure and found that the straw increased the relative abundance of Ascomycota and Mortierellomycota. In conclusion, compared with no straw return, wheat straw return significantly increased the relative abundance of Ascomycota in the first season and decreased the relative abundance of Ascomycota in the second season. Combining the LEfSe analysis with microbial community composition, we detected pathogenic genera in the soil microbial community after different straw return treatments of wheat and maize, such as *Fusarium*, a soil-borne pathogenic fungal genus that causes outbreaks of fungal diseases such as wheat scab, further harming human health (Zhao et al., 2014; Kumar et al., 2018). Compared with no straw return, wheat straw return increased the relative abundance of *Fusarium* in both seasons, suggesting that wheat straw return increased the possibility of soil-borne disease outbreaks. Therefore, appropriate agricultural measures should be taken, such as crop rotation and straw return, to reduce the incidence of disease. However, Tang et al. (2020) found that, for fungal microorganisms, the relative abundance of *Fusarium* decreased with the addition of wheat straw, which was different from the results of this study. At the same time, based on the results of the microbial network analysis, they showed that fungal communities have more complex relationships than bacterial communities. The symbiotic network analysis in this study showed that the symbiotic relationships of bacteria in the soil were more dominant than those of fungi. In addition, compared with no straw return, the first season straw return increased the relative abundance of *Chaetomium*. However, only SB increased the relative abundance of *Chaetomium*, whereas SW and SM decreased it. Recent studies showed that *Chaetomium* is a saprophyte fungus that participates in the degradation of organic matter (Rice and Currah, 2006). Compared with no straw return, FM decreased the relative abundance of *Trichoderma*, while SM increased it. *Trichoderma* is antagonistic to many plant-pathogenic fungi and can promote plant growth and crop yield (Alwadai et al., 2022). These results indicate that long-term straw return accelerates the conversion of soil organic matter, which affects the contents of harmful and beneficial microorganisms in the soils, thus changing the soil microbial community structure.

## Conclusion

Our results showed that straw returning before wheat or maize crops has different effects on soil microbial diversity and community structure but promotes the proliferation of bacteria or fungal microorganisms in certain soils. We found that straw return significantly increased the relative abundance of Ascomycota in the first season and decreased the relative abundance of Ascomycota in the second season. However, in the second season, wheat straw return increased the relative abundance of *Bradyrhizobium*, a bacteria related to the soil nitrogen cycle. In addition, we detected two fungal pathogens (*Fusarium* and *Trichoderma*) under the two planting patterns and found that the relative abundance of pathogenic *Fusarium* increased with wheat straw return (FW and SW). *Trichoderma* increased after maize straw return before wheat planting (S group). Altogether, these results suggest that wheat straw return (FW and SW) and maize straw return might have a negative impact on pathogenic risk. Therefore, further studies are needed to determine how to manage straw returns in agricultural production.

## Data availability statement

The datasets presented in this study can be found in online repositories. The names of the repository/repositories and accession number(s) can be found in the article/Supplementary material.

## Author contributions

YP and SZ planned and designed the project. SZ and ML performed the experiments. SZ, ML, and XC analyzed the data and wrote the manuscript. All authors discussed the results and contributed to the final manuscript.
