# Supplementary material for: Effect of different straw retention techniques on soil microbial community structure in wheat–maize rotation system
Source: Front Microbiol. 2023 Jan 19;13:1069458. doi: 10.3389/fmicb.2022.1069458 (PMC9893011; doi:10.3389/fmicb.2022.1069458)
Supplement: Supplementary file 1 [file Data_Sheet_1.PDF]

Supplementary material

Effect of different straw retention on Soil Microbial Community Structure in wheat-corn rotation system

Shulin Zhang[1, 2#], Meng Li[1, 2#], Xinyue Cui[1, 2#], Yuemin Pan[1, 2*]

1. Department of Plant Pathology, College of Plant Protection, Anhui Agricultural University, Hefei, Peoples' Republic of China 230036.

2. Anhui Province Key Laboratory of Crop Integrated Pest Management, Anhui Agricultural University, Hefei, Peoples' Republic of China 230036.

# These authors contributed equally to this work

* Address correspondence to YM Pan (panyuemin2008@163.com)

This PDF file includes

Figure and Table

Figure. S1 Phylum level community composition of bacteria based on 16S rRNA gene and fungi based on ITS gene in different straw returning treatment groups of group F (Note: In order to achieve the best view effect, the parts with abundance less than 1% can be merged as other in the diagram).

S1

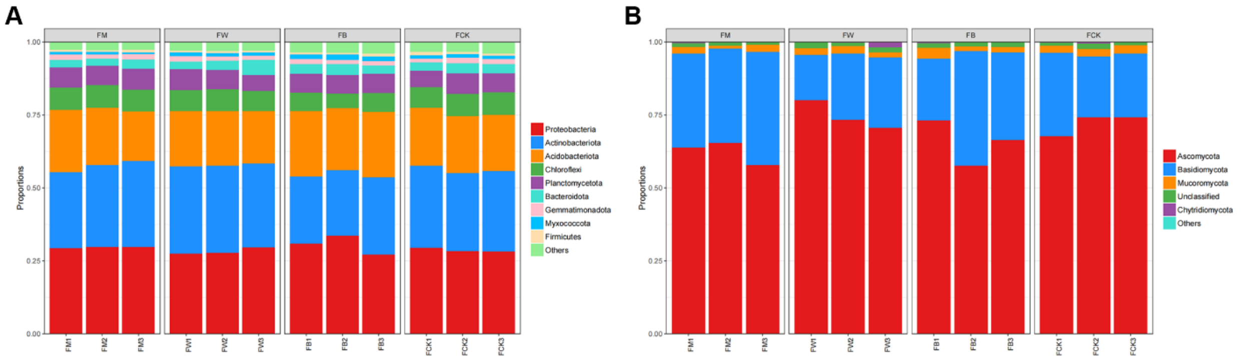

Figure. S2 Phylum level community composition of bacteria based on 16S rRNA gene and fungi based on ITS gene in different straw returning treatment groups of group S (Note: In order to achieve the best view effect, the parts with abundance less than 1% can be merged as other in the diagram).

S2

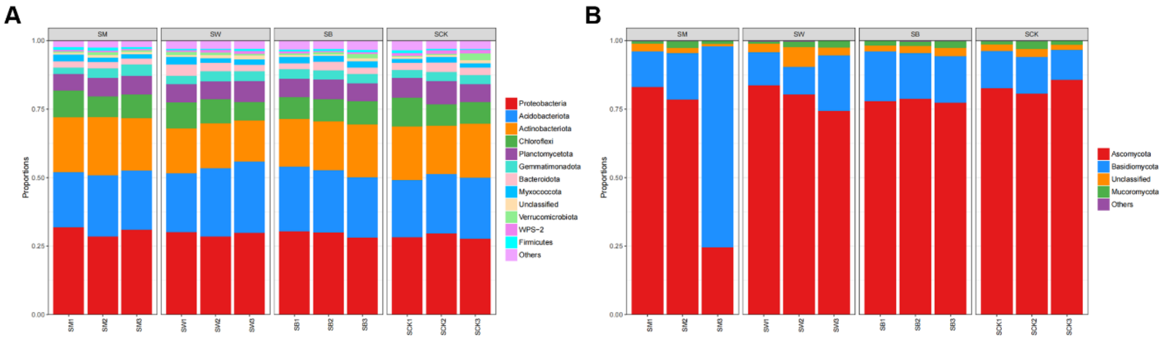

Figure. S3 Cladogram plotted from LEfSe analysis showing the significant differences (P < 0.05) in relative abundance of 16S rRNA gene-based bacterial taxon among four treatments before wheat planting (A). Results of LEfSe analysis showing taxa that significantly differed in the four treatments before wheat planting (B). Cladogram plotted from LEfSe analysis showing the significant differences (P < 0.05) in relative abundance of ITS gene-based Fungi taxon among four treatments before wheat planting (C). Results of LEfSe analysis showing taxa that significantly differed in the four treatments before wheat planting (D)

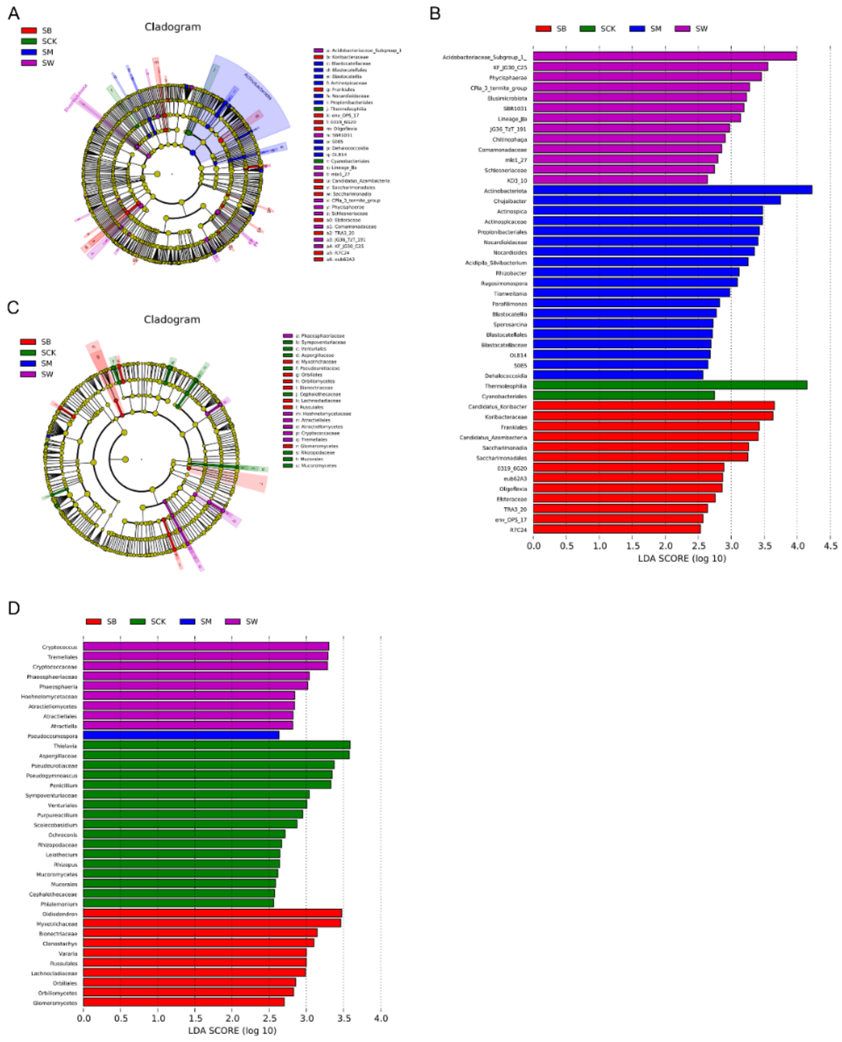

Table S1 The relative abundance of phyla and genera (top 10) for 16S rRNA gene in group F. The relative abundance of phyla and genera (top 10) for 16S rRNA gene. The asterisk (*) and (**) that follows the taxonomic name represents significant differences (P < 0.05) and (P < 0.01) among the four treatments, respectively.

Classification level	Top 10 bacteria	P-value	Relative abundance			
			FM	FW	FB	FCK
Phylum	Proteobacteria	0.3806	0.2965	0.2829	0.3062	0.2872
	Actinobacteriota*	0.0554	0.2784	0.2955	0.2399	0.2752
	Acidobacteriota**	0.0050	0.1931	0.1859	0.2198	0.1952
	Chloroflexi*	0.0272	0.0764	0.0716	0.0594	0.0752
	Planctomycetota	0.7599	0.0693	0.0640	0.0648	0.0635
	Bacteroidota	0.5506	0.0271	0.0359	0.0334	0.0311
	Gemmatimonadota	0.5162	0.0175	0.0164	0.0153	0.0167
	Myxococcota*	0.0331	0.0076	0.0123	0.0164	0.0118
	Firmicutes	0.6949	0.0066	0.0058	0.0076	0.0085
	others	-	-	-	-	-
Genus	*Gaiellales_norank**	0.0399	3318.6667	2889.0000	2391.3333	3159.0000
	Acidobacteriales_norank	0.0705	1438.3333	1333.3333	1185.0000	1595.3333
	Bradyrhizobium	0.2086	889.0000	843.6667	941.6667	851.6667
	*JG30-KF-AS9_norank***	0.0121	998.0000	840.0000	613.0000	930.3333
	Acidibacter	0.8634	870.0000	739.6667	872.6667	847.0000
	*Micropepsaceae_uncultured***	0.0122	723.3333	637.0000	920.6667	738.6667
	*Occallatibacter***	0.0199	620.3333	767.3333	871.0000	677.6667
	Granulicella	0.1898	738.3333	622.3333	824.6667	654.3333
	*67-14_norank**	0.0282	494.6667	645.6667	614.3333	461.3333
	Bryobacter	0.3640	499.3333	571.3333	555.6667	521.6667

Table S2 The relative abundance of phyla and genera (top 10) for ITS gene in group F. The asterisk (*) and (**) that follows the taxonomic name represents significant differences ($P < 0.05$) and ($P < 0.01$) among the four treatments, respectively.

Classification level	Top Fungi	P-value	Relative abundance			
			FM	FW	FB	FCK
Phylum	Ascomycota*	0.0388	0.6241	0.7476	0.6574	0.7206
	Basidiomycota*	0.0330	0.3437	0.2074	0.3018	0.2380
	Mucoromycota	0.1987	0.0195	0.0220	0.0241	0.0262
	Chytridiomycota	0.5126	0.0016	0.0067	0.0015	0.0027
Genus	*Chaetomium*	0.1382	8336.0000	7754.0000	9123	7225.6667
	Trechispora	0.3013	8108.0000	3600.6667	6971.6667	4333.6667
	Solicoccozyma	0.0625	1631.0000	1832.6667	1155.3333	1801
	Podospora	0.2063	1009.0000	1791.6667	1008	1361.6667
	Fusarium	0.4637	1189.3333	1382.0000	1106.3333	1272.6667
	Humicola	0.0947	719.3333	992.6667	733.6667	1852.6667
	Trichoderma	0.6462	865.0000	1232.0000	909.6667	1076.6667
	Mortierella	0.5638	506.3333	650.3333	649.6666667	755
	Exophiala	0.4969	435.0000	811.3333	600	703.3333333
	*Cladophialophora**	0.0309	386.6667	444.3333	650.6666667	346.3333333

Table S3 The relative abundance of phyla and genera (top 10) for 16S rRNA gene in group S. The asterisk (*) and (**) that follows the taxonomic name represents significant differences ($P < 0.05$) and ($P < 0.01$) among the four treatments, respectively.

Classification level	Top 10 bacteria	P-value	Relative abundance			
			SM	SW	SB	SCK
Phylum	Proteobacteria	0.3334	0.3046	0.2952	0.2951	0.2852
	Acidobacteriota	0.1274	0.2138	0.2412	0.2282	0.2166
	Actinobacteriota	0.3532	0.2015	0.1593	0.1813	0.1902
	Chloroflexi	0.6543	0.0861	0.0834	0.0821	0.0867
	Planctomycetota	0.3873	0.0656	0.0691	0.0680	0.0742
	Gemmatimonadota	0.2252	0.0327	0.0342	0.0341	0.0315
	Bacteroidota	0.3324	0.0224	0.0324	0.0249	0.0293
	Myxococcota*	0.0297	0.0196	0.0215	0.0230	0.0173
	Verrucomicrobiota	0.8340	0.0059	0.0083	0.0094	0.0111
	WPS-2*	0.0276	0.0062	0.0088	0.0061	0.0114
Genus	Gaiellales_norank**	0.0043	2743.6667	2410.6667	2625.6667	2952.6667
	Acidobacteriales_norank	0.0682	1882.0000	1933.6667	1862.0000	2207.6667
	JG30-KF-AS9_norank	0.9081	1150.0000	1035.6667	1062.0000	1021.3333
	Micropepsaceae_uncultured	0.9618	1018.0000	1084.0000	1050.6667	1080.0000
	Acidibacter*	0.0371	1100.6667	1114.0000	1036.6667	860.3333
	Occallatibacter**	0.0184	546.6667	922.3333	598.6667	672.6667
	Gemmatimonadaceae_uncultured	0.4787	661.0000	715.0000	712.3333	637.3333
	Acidobacteriaceae (Subgroup 1)_uncultured**	0.0068	503.6667	711.0000	824.6667	654.3333
	Bradyrhizobium	0.8060	626.0000	676.6667	614.3333	461.3333
	Bryobacter	0.2657	622.6667	629.3333	555.6667	521.6667

Table S4 The relative abundance of phyla and genera (top 10) for ITS gene in group S. The asterisk (*) and (**) that follows the taxonomic name represents significant differences (P < 0.05) and (P < 0.01) among the four treatments, respectively.

Classification level	Top Fungi	P-value	Relative abundance			
			SM	SW	SB	SCK
Phylum	Ascomycota*	0.0319	0.6203	0.7950	0.7806	0.8301
	Basidiomycota**	0.0096	0.3449	0.1411	0.1723	0.1262
	Mucoromycota	0.9759	0.0152	0.0177	0.0187	0.0185
Genus	Chaetomium	0.7256	5986.0000	10150.3333	11636.3333	10912.0000
	Solicoccozyma	0.7432	1974.3333	3024.6667	2708.6667	2854.6667
	Trechispora	0.3398	7851.6667	787.6667	120.0000	365.3333
	Podospora	0.4663	2114.3333	2020.0000	1882.6667	1541.0000
	Fusarium	0.5760	1992.6667	1340.6667	1259.6667	1156.6667
	Humicola*	0.0284	1005.3333	1813.3333	1033.3333	1755.3333
	Trichoderma	0.5467	1166.0000	1371.0000	1196.0000	1073.0000
	Coniochaeta	0.5245	269.0000	1241.0000	988.6667	1170.3333
	Talaromyces	0.1570	3059.6667	116.3333	267.3333	140.0000
